# Effects of Traditional Chinese Medicine and its Active Ingredients on Drug-Resistant Bacteria

**DOI:** 10.3389/fphar.2022.837907

**Published:** 2022-06-02

**Authors:** Jimin Li, Shanshan Feng, Xin Liu, Xu Jia, Fengling Qiao, Jinlin Guo, Shanshan Deng

**Affiliations:** ^1^ Chongqing Key Laboratory of Sichuan-Chongqing Co-construction for Diagnosis and Treatment of Infectious Diseases Integrated Traditional Chinese and Western Medicine, College of Medical Technology, Chengdu University of Traditional Chinese Medicine, Chengdu, China; ^2^ Non-Coding RNA and Drug Discovery Key Laboratory of Sichuan Province, Chengdu Medical College, Chengdu, China; ^3^ School of Basic Medical Sciences, Chengdu Medical College, Chengdu, China; ^4^ School of Public Health, Chengdu Medical College, Chengdu, China; ^5^ Key Laboratory of Systematic Research of Distinctive Chinese Medicine Resources in Southwest China, Chengdu University of Traditional Chinese Medicine, Chengdu, China

**Keywords:** traditional Chinese medicine, active ingredient, combined, antibiotic, drug-resistant bacterial

## Abstract

The increasing and widespread application of antibacterial drugs makes antibiotic resistance a prominent and growing concern in clinical practice. The emergence of multidrug-resistant bacteria presents a global threat. However, the development and use of novel antibacterial agents involves time-consuming and costly challenges that may lead to yet further drug resistance. More recently, researchers have turned to traditional Chinese medicine to stem the rise of antibiotic resistance in pathogens. Many studies have shown traditional Chinese medicines to have significant bacteriostatic and bactericidal effects, with the advantage of low drug resistance. Some of which when combined with antibiotics, have also demonstrated antibacterial activity by synergistic effect. Traditional Chinese medicine has a variety of active components, including flavonoids, alkaloids, phenols, and quinones, which can inhibit the growth of drug-resistant bacteria and be used in combination with a variety of antibiotics to treat various drug-resistant bacterial infections. We reviewed the interaction between the active ingredients of traditional Chinese medicines and antibiotic-resistant bacteria. At present, flavonoids and alkaloids are the active ingredients that have been most widely studied, with significant synergistic activity demonstrated when used in combination with antibiotics against drug-resistant bacteria. The reviewed studies show that traditional Chinese medicine and its active ingredients have antimicrobial activity on antibiotic-resistant bacteria, which may enhance the susceptibility of antibiotic-resistant bacteria, potentially reduce the required dosage of antibacterial agents and the rate of drug resistance. Our results provide direction for finding and developing alternative methods to counteract drug-resistant bacteria, offering a new therapeutic strategy for tackling antibiotic resistance.

## Introduction

In the late 1950s, most *Staphylococcus aureus* strains became resistant to penicillin (Paul D Stapleton, 2002). Researchers then developed new drugs, such as methicillin and vancomycin, to treat penicillin-resistant bacteria. Unfortunately, the existence of methicillin-resistant *S. aureus* (MRSA) was first reported in 1961 (Barber, 1961). Antibiotic resistance is a global problem. Although it is a natural process for bacteria to develop antibiotic resistance, antibiotic resistance is accelerated by the misuse and abuse of antibiotics, which makes it more difficult to prevent and control bacterial infections (Piddock, 2012). Currently, more and more infections become complicated to treat or even untreatable, as overuse of antibiotics reduces their effectiveness. Thus far, there is no antibiotic capable of solving the problem of resistant strains, where it is predicted that antibiotic resistance will re-emerge even with the most vigorous research and development of new drugs (Barriere, 2014). Antibiotic resistance leads to higher hospital costs, delayed discharge times and higher mortality rates, where at least 700,000 people die worldwide each year as a result. The report on the review of Antimicrobial Resistance chaired by Jim O’Neill warns that if bacterial drug resistance remains to increase at the rate of today’s levels, 10 million people per year may die of antibiotic resistance by 2050.

In recent years, the exploration of methods to control drug-resistant strains has attracted extensive attention from scholars hoping to find a promising alternative solution. Traditional Chinese medicine (TCM) has attracted the greatest interest among all methods. TCM has a long history and rich experience in treating infectious diseases. The antibacterial action of TCM and its compounds has a complex multi-link, multi-target, and multi-site process. Compared with antibiotics, TCM is characterised with more resources, easier access, lower drug resistance, more active ingredients (Yang et al., 2010; Wu et al., 2019) fewer adverse reactions, and more targets (Messier and Grenier, 2011; Eumkeb et al., 2012a). Many studies have shown that TCM has significant bacteriostatic or bactericidal effects. These effects occur mainly through inhibition of biofilm formation of drug-resistant bacteria, efflux pump system, enzyme activity, and changes in the permeability of bacteria and other drug-resistant mechanisms (Su et al., 2020). *Polygonum cuspidatum* (*Polygonum cuspidatum* Sieb. et Zucc.) extracts can exert antibacterial and bactericidal effects by destroying bacterial cell membranes and walls (Su et al., 2015). Extracts from *Hypericum perforatum* (*Hypericum perforatum* L.) and *Sophora moorcroftiana* (*Sophora moorcroftiana* (Benth.Baker)) also have antibacterial effects, as the extracts can inhibit the growth of drug-resistant bacteria by suppressing the efflux pump system (Wang et al., 2014; Dogan et al., 2019). Resveratrol can inhibit biofilm formation of avian pathogenic *Escherichia coli* to achieve a bacteriostatic effect (Ruan et al., 2021).

**FIGURE 1:**
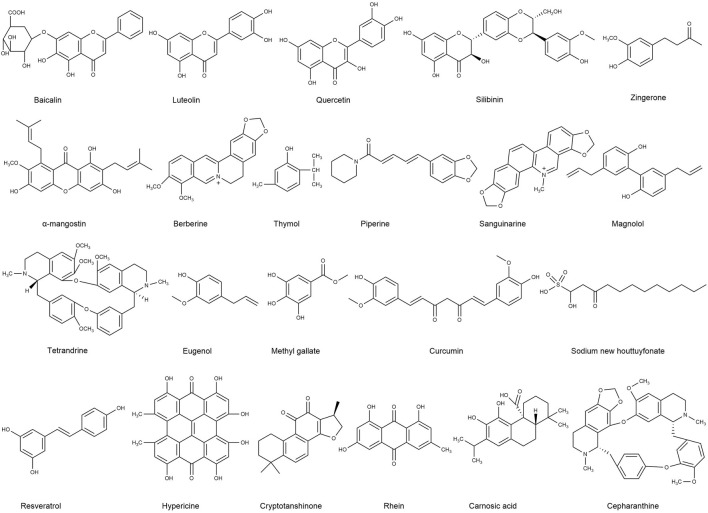
List of key compounds studied for their synergy with antibiotics.

Studies have demonstrated that some TCM can directly inhibit drug-resistant bacteria. However, for TCM with no individually attributed antibacterial activity, if combined with antibacterial drugs, the synergistic effect of TCM can make these TCM play an important role in bacterial infection treatment. The synergistic effect by TCM can also enhance the susceptibility of drug-resistant bacteria to antibiotics and even reverse drug resistance. Studies on the antibacterial effects of pterostilbene and gentamicin alone and in combination showed no significant difference in antibacterial effects. However, when they were combined they completely inhibited the growth of bacteria and had synergistic antibacterial effects (Lee et al., 2017). The synergistic application of TCM and antibiotics in drug-resistant bacteria has stronger antibacterial activity, which is a recognised antibacterial treatment measure (Wagner and Ulrich-Merzenich, 2009). Several alternative antibiotic treatments for bacteria, such as bacteriocins (Cotter et al., 2013), essential oils (Esmael et al., 2020; Puvaca et al., 2021), antibodies (Berghman et al., 2005), and phage therapy (Chang et al., 2018), have been evaluated in studies and confirmed *in vitro* and with the use of animal models. However, these still present with many issues to consider, including cost, side effects, and safety, where most of them are still far from clinical use. As TCM has already been used clinically with a long history, combining antibiotics and TCM is a promising alternative therapy to resolve antibiotic resistance. As extracts from TCM may contain hundreds of chemical components, the isolation of active compounds under the guidance of bioassays is crucial to study their synergistic effects in detail. This review summarises the effects of flavonoids, alkaloids, phenols, and quinones (chemical structures of key compounds in these classes are shown in Figure 1) combined with antibiotics on bacterial and drug-resistant bacterial infections. It provides the basis for an alternative approach, involving TCM to treat bacterial and drug-resistant bacterial infections in the future, by applying a relatively new and promising option in antibiotic resistant treatment.

## Methodology

Search strategy and research criteria: English articles published from September 2001 to May 2021 were searched in the PubMed database, and related keywords such as: “Traditional Chinese medicine,” “Chinese herbal medicine,” “antibiotics,” “drug-resistant bacteria,” “flavonoids,” “alkaloids,” “phenols,” and “quinones” were used to search the database. The study included published data but excluded TCM treatments for other diseases, such as cancer. 180 English language articles published mainly since 2011 were located which related to the use of components from TCM against drug-resistant bacteria. According to our criteria, we reviewed the abstract and content of the articles, with 115 studies included as references, among which 86 were identified. Most of these papers focus on the synergistic antibacterial activity of the active ingredients of TCM combined with antibiotics against drug-resistant bacteria, and how some active ingredients of TCM can reverse drug resistance.

Synergy judgment criteria: In order to assess if a TCM component in combination with an antibiotic demonstrated a synergistic activity, we used the published definition of the fractional inhibitory concentration index (FICI), which is the sum of the FICs of each of the drugs, which were defined as the minimal inhibition concentration (MIC) of each drug when used in combination divided by the MIC of each drug when used alone, i.e., FICI = (MIC of drug A in combination/MIC of drug A alone) + (MIC of drug B in combination/MIC of drug B alone). FICI were graded as: ≤ 0.5, synergy; > 0.5–≤ 1.0, additive; >1.0–≤ 2.0, indifference; and >2.0, antagonism (Kang et al., 2011).

## Review

### Flavonoids Combined With Antibiotics for Antibacterial Effects

Flavonoids are compounds of some widely distributed plants and are found in photosynthetic cells, which exist broadly within the plant kingdom and in almost all parts of the plant (Havsteen, 1983). Baicalein and baicalin in the root of *Scutellaria baicalensis* Georgi, luteolin in the root and stem of *Reseda odorata* L*.*, and quercetin in the flower and leaf of *Camellia sinensis* (L.) Kuntze are all flavonoids. For centuries, preparations containing flavonoids as the key physiologically active ingredients have been used by clinicians to treat human diseases. It is reported that flavonoids have anti-inflammatory and antibacterial effects, whilst potentially having antiviral, antioxidant and free radical scavenging abilities (Kumar and Pandey, 2013). Researchers have also actively investigated the antibacterial effects of flavonoids in combination with antibiotics.

**FIGURE 2:**
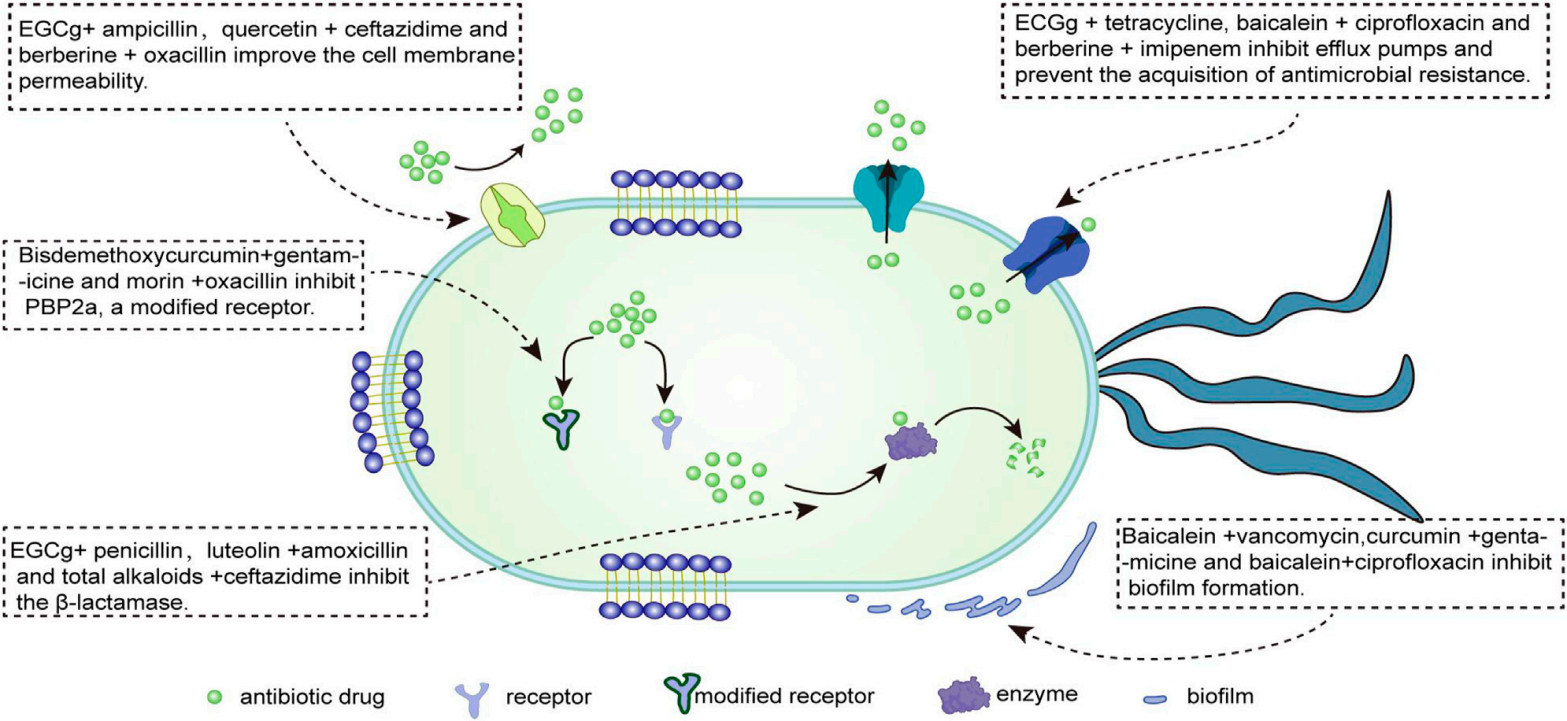
The major mechanism and target of the antibacterial effect of antibiotics combined with active ingredients of traditional Chinese medicine.

Mai Fujita et al. (2005) demonstrated that the combination of baicalein with tetracycline and β-lactam antibiotics significantly reduced the MIC of MRSA such that it played an antibacterial role. When baicalein and ciprofloxacin were combined to treat MRSA infection, 12 of the 20 drug-resistant strains had FICI≤0.5, which mainly inhibited the efflux of ciprofloxacin by suppressing the efflux pump, thereby exerting a synergistic anti-MRSA effect (Chan et al., 2011). The main mechanism of the combination of active ingredients of TCM and antibiotics is shown in Figure 2. Qian et al. (2015) also found that the combined application of baicalein and penicillin can resist penicillinase-producing MRSA or *S. aureus* infection. When the concentration of baicalein increased from 8 μg/ml to 32 μg/ml, the MIC of penicillin decreased from 64 μg/ml to 4 μg/ml, significantly improving the resistant bacteria’s susceptibility to penicillin. Recent studies have demonstrated that linezolid and baicalein can inhibit biofilm formation *in vivo* to play an anti-MRSA role (Liu T. et al., 2020). Baicalin has similar effects to baicalein, and if Baicalin is used in combination with oxytetracycline and tetracycline, it can resist *S. aureus* infection, while in combination with β-lactam antibiotics, it yields anti-MRSA activity (Iain and Liu, 2000; Novy et al., 2011).

Usman Amin et al. (2016) demonstrated synergistic effects of luteolin and quercetin combined with ceftriaxone and imipenem against MRSA. In addition, luteolin combined with ampicillin, oxacillin, and gentamicin can synergically enhance the antibacterial action of aminoglycosides and β-lactam antibiotics against MRSA. The FICI of the combination of 12 MIC luteolin and 12 MIC antibiotics against MRSA ATCC 33591 for most strains was 0.125–0.562, and these combinations did not show additive or antagonistic effects (Joung et al., 2016). As well as inhibiting MRSA, luteolin can synergize with amoxicillin to reverse the resistance of amoxicillin-resistant *E. coli* and can fight *Streptococcus pyogenes* infection when combined with ceftazidime. Quercetin can also combat *S. pyogenes* combined with ceftazidime, where the FICIs of luteolin and quercetin paired with ceftazidime were 0.37 and 0.27, respectively (Eumkeb et al., 2012b; Siriwong et al., 2015). Siriwong et al. (2016) also demonstrated that quercetin with amoxicillin could reverse the resistance of amoxicillin-resistant *Staphylococcus epidermidis*. In addition, quercetin with ciprofloxacin, tetracycline, and erythromycin has an antibacterial effect on *S. aureus,* including MRSA. In the time-kill curves test, quercetin with tetracycline reduced the cell viability of resistant *E. coli* strains by more than eight times within 24 h compared with the drug group alone and had a FICI ≤0.5 (Abreu et al., 2016; Qu et al., 2019). Compared with other antibiotics, researchers found that ¼ MIC, 18 MIC quercetin combined with tobramycin and amikacin has potential systematic antibacterial activity against multidrug-resistant *Pseudomonas aeruginosa* (Vipin et al., 2020). Pal and Tripathi (Pal and Tripathi, 2019; 2020) reported that quercetin and meropenem had synergistic antibacterial effects on carbapenem-resistant *P. aeruginosa*, *A. baumannii*, *E. coli,* and *K. pneumoniae*, with FICI values of 0.18–0.50, 0.16–0.37, 0.187–0.375, and 0.093–0.500, respectively, which can not only significantly kill bacteria but also may reverse drug resistance.

It has been reported (Kang et al., 2011; Cai et al., 2018; Vivekanandan et al., 2018) that silibinin, an extract of *Silybum marianum* (L.) Gaertn., has anti-MRSA activity when combined with oxacillin or ampicillin. Another extract, silymarin, can improve the toxicity of linezolid and synergistic anti-MRSA infection, while a high concentration silibinin with kanamycin can inhibit the growth of *S. aureus.* Pimchan et al. (2017) demonstrated a synergistic effect between α-mangostin and ceftazidime in *A. baumannii*. The FICI of the combination of α-mangiferin and oxacillin against oxacillin-resistant *Staphylococcus saprophyticus* was 0.37. The number of bacterial colonies decreased by the combination of 2 μg/ml α-mangostin and 16 μg/ml oxacillin, and in the time-kill curves test ≥2 log10 cfu/ml also verified the synergy. When α-mangostin is combined with gentamicin and vancomycin hydrochloride, it can help inhibit vancomycin-resistant *Enterococci* (VRE) and MRSA infection, respectively (Sakagami et al., 2005; Phitaktim et al., 2016). Table 1 lists the antibacterial effects of flavonoids combined with antibiotics.

**TABLE 1:** Summary of flavonoids compounds in combination with antibiotics.

Species Name	Active ingredients	Drug resistant strains	Combination antibiotics	FICI	References
*Thymus vulgaris* L.	Baicalein	MRSA	Tetracycline, β -lactam antibiotics	—	Mai Fujita et al. (2005)
*Scutellaria baicalensis* Georgi	Baicalein	MRSA	Ciprofloxacin	≤0.5	Chan et al. (2011)
*Scutellaria baicalensis* Georgi	Baicalein	MRSA	Linezolid	—	Liu et al. (2020a)
*Scutellaria baicalensis* Georgi	Baicalein	*MRSA*,*Staphylococcus aureus*	penicillin	0.14–0.38	Qian et al. (2015)
*Scutellaria baicalensis* Georgi	Baicalin	*Staphylococcus* aureus	Oxytetracycline, Tetracycline	≤0.5	(Iain and Liu, 2000; Novy et al. (2011)
*Scutellaria amoena* C.H. Wright	Baicalin	MRSA	β -lactam antibiotics	≤0.5	-
*Lonicera japonica* Thunb., *Thymus vulgaris* L.	Luteolin	MRSA	Ceftriaxone, Imipenem	0.45–0.50	Usman Amin et al. (2016)
*Thymus vulgaris* L., *Daucus carota* L.	Luteolin	MRSA	Ampicillin, Oxacillin, Gentamicin	0.125–0.562	Joung et al. (2016)
*Thymus vulgaris* L., *Daucus carota* L.	Luteolin	*Escherichia coli*	Amoxicillin	≤0.5	Eumkeb et al. (2012b); Siriwong et al. (2015)
*Daucus carota* L., *Allium cepa* L.	Luteolin, Quercetin	*streptococcus pyogenes*	Ceftazidime	0.37、0.27	-
*Allium cepa* L., *Ginkgo biloba* L.	Quercetin	*Staphylococcus epidermidis*	Amoxicillin	0.5	Siriwong et al. (2016)
*Allium cepa* L., *Ginkgo biloba* L.	Quercetin	MRSA	Ciprofloxacin, Tetracycline and Erythromycin	—	(Abreu et al., 2016; Qu et al., 2019)
*Allium cepa* L., *Ginkgo biloba* L.	Quercetin	*Escherichia coli*	Tetracycline	≤0.5	-
*Allium cepa* L., *Ginkgo biloba L.*	Quercetin	*pseudomonas aeruginosa*	Tobramycin, Amikacin	0.25–0.5	Vipin et al. (2020)
*Allium cepa* L., *Berberis aristata* DC., *Camellia sinensis* (L.) Kuntze	Quercetin	*Pseudomonas aeruginosa, Acinetobacter baumannii, Escherichia coli, Klebsiella pneumoniae*	Meropenem	0 .18-0.5、0.16-0 .37、0.187-0.375和0.093-0.5	Pal and Tripathi, (2019); Pal and Tripathi, (2020)
*Silybum marianum* (L.) Gaertn.	Silibinin	MRSA	Oxacillin, Ampicillin	≤0.5	Kang et al. (2011); Cai et al. (2018); Vivekanandan et al. (2018)
*Silybum marianum* (L.) Gaertn.	Silibinin	*Staphylococcus aureus*	Kanamycin	—	-
*Silybum marianum* (L.) Gaertn.	Silymarin	MRSA	Linezolid	—	-
*Garcinia mangostana* L.	α-Mangostin	*Acinetobacter Baumannii*	Ceftazidime	<0.35	Pimchan et al. (2017)
*Garcinia mangostana* L.	α-Mangostin	*Staphylococcus saprophytic*	Oxacillin	0.37	Sakagami et al. (2005); Phitaktim et al. (2016).
*Garcinia mangostana* L.	α-Mangostin	*Enterococcus*, MRSA	Gentamicin, Vancomycin hydrochloride	≤0.5	-

### Alkaloids Combined With Antibiotics for Antibacterial Effects

Alkaloids are components of botanical drugs and are widely distributed in nature. They are organic compounds with biological activity and are present within a wide range of plants, bacteria, and fungi (Qiu et al., 2014). Berberine is extracted from *Berberis vulgaris* L., total alkaloids from *Sophora alopecuroides* L., and tetrandrine from *Stephania tetrandra* S. Moore are common alkaloids. Several clinical studies have reported that alkaloids have anti-inflammatory (Souza et al., 2020), antibacterial activities (Liu Y. et al., 2020) and antiviral (Gorpenchenko et al., 2019) pharmacological effects. Studies have shown that these alkaloid compounds are important in enhancing antibiotic effects for treating infections (Cushnie et al., 2014). In recent years, researchers have explored cooperative applications of alkaloids and antibiotics to fight against bacterial resistance.

Hyeon-Hee et al. (2005) showed the anti-MRSA effect of berberine. The FICI of berberine combined with ampicillin (0.625) had an additive effect, whereas if it joined with oxacillin (0.5) it had a synergistic effect. Some scholars have found that berberine combined with azithromycin has a synergistic antibacterial effect on MRSA and *P. aeruginosa*, and if it paired with levofloxacin, it could resist MRSA infection. The combination of ¼ MIC berberine and 18 MIC imipenem had a synergistic antibacterial effect on carbapenems resistant *P. aeruginosa* with a FICI of 0.375. In addition, berberine can increase the antibacterial activity of gentamicin and other aminoglycoside antibiotics against *P. aeruginosa* and reverse the resistance of antibacterial drugs. When berberine was combined with linezolid, cefoxitin, and erythromycin, the synergistic effect was significant in coagulase-negative *staphylococcus* (Zuo et al., 2012; Wojtyczka et al., 2014; Morita et al., 2016; Li et al., 2017; Su and Wang, 2018). Although the FICI of berberine and ciprofloxacin against multidrug-resistant *Salmonella* and *K. pneumoniae* were between 0.375 and 1, the time-kill curves test confirmed the synergistic antibacterial effect of the combination (Zhou et al., 2016; Shi et al., 2018). Studies have shown that berberine and fluconazole can be combined to resist drug-resistant *Candida albicans* and fluconazole-resistant *Candida tropicalis*. Berberine can increase the biosynthesis of ergosterol, making it resistant to *C. albicans*. The effect of fluconazole on ergosterol can eliminate the resistance of berberine and synergise with berberine against drug-resistant *C. albicans*. Berberine and fluconazole also synergise against fluconazole-resistant *Candida tropicalis* by inhibiting efflux pumps (Shi et al., 2017; Xu et al., 2017). Liang et al. (2014) showed that an isoquinoline alkaloid may be extracted from *Berberis vulgaris* L. and other plants. The combination of berberine chloride and fusidic acid has shown a synergistic antibacterial effect on seven clinically isolated MRSA strains, with most significant inhibitions on two highly resistant strains, 4,806 and 7,155-1, and their FICIs were 0.19 and 0.38, respectively. Berberine chloride can increase the susceptibility of multidrug-resistant *A. baumannii* to tigecycline, sulbactam, meropenem, and ciprofloxacin to facilitate a more effective antibacterial role (Li et al., 2021). When berberine chloride combined with penicillin, clindamycin, and erythromycin, can also significantly inhibit the growth of *Streptococcus oralis* in a dose-dependent manner. Further, when combined with vancomycin, it can greatly inhibit the growth and motor capacity of *Clostridium difficile,* and can synergistically inhibit drug-resistant *C. albicans* when paired with fluconazole (Dziedzic et al., 2015; Wultanska et al., 2020; Yong et al., 2020).

Khameneh et al. (2015) demonstrated that the co-application of piperine and gentamicin nanoliposomes on MRSA had a significant synergistic antibacterial effect. Some researchers have shown that low-dose total alkaloids of *Sophora alopecuroides* L. and ciprofloxacin have synergistic antibacterial activity against multidrug-resistant *E. coli*. Total alkalids can enhance bacterial susceptibility to ciprofloxacin and cooperate with cefotaxime and ceftazidime against extended-spectrum β-lactamase (ESBL)-producing *E. coli* infection (Zhou et al., 2013; Pourahmad Jaktaji and Mohammadi, 2018). In time-kill curve tests, Zhang et al. (2010) showed that the combined application of 30 μg/ml tetrandrine and ketoconazole on drug-resistant *Candida* had synergistic antibacterial effects *in vitro* and *in vivo* but had no bactericidal effect. Tetrandrine and cefazolin in bisbenzylisoquinoline alkaloids presented a considerable synergistic effects against 90% of 10 clinically isolated MRSA strains, with the FICI between 0.188 and 0.625, while demethyltetrandrine and cefazolin had respective additive activities against 50% and 90% of tested MRSA strains, with the FICI ranging from 1.5 to 2.0 (Zuo et al., 2011). Another compound from TCM, called sanguinarine, can restore antibacterial activity of ampicillin, oxacillin, norfloxacin, and ciprofloxacin to treat MRSA by inhibiting the growth of drug-resistant bacteria (Obiang-Obounou et al., 2011). Table 2 lists the antibacterial effects of the above alkaloids combined with antibiotics.

**TABLE 2:** Summary of alkaloids compounds in combination with antibiotics.

Species Name	Active ingredients	Drug resistant strains	Combination antibiotics	FICI	References
*Coptis chinensis* Franch., *Phellodendron amurense* Rupr.	Berberine	MRSA	Oxacillin	0.5	Hyeon-Hee Yu (2005)
*Coptis chinensis* Franch., *Phellodendron amurense* Rupr.	Berberine	MRSA	Azithromycin, Levofloxacin	0.188–0.5	Zuo et al. (2012); Wojtyczka et al. (2014); Morita et al. (2016); Li et al. (2017); Su and Wang, (2018)
*Coptis chinensis* Franch., *Phellodendron amurense* Rupr.	Berberine	*Pseudomonas aeruginosa*	Azithromycin	0.13–0.5	-
*Coptis chinensis* Franch., *Phellodendron amurense* Rupr.	Berberine	*Pseudomonas aeruginosa*	Gentamicin and other aminoglycoside antibiotics	<0.5	-
*Coptis chinensis* Franch., *Phellodendron amurense* Rupr.	Berberine	*Pseudomonas aeruginosa*	Imipenem	0.375	-
*Coptis chinensis* Franch., *Berberis vulgaris* L., *Berberis aristate* DC	Berberine	Coagulase negative *staphylococcus*	Linezolid, Cefoxitin and Erythromycin	—	-
*Coptis chinensis* Franch.	Berberine	*Salmonella, Klebsiella pneumoniae*	Ciprofloxacin	0.375–1	Zhou et al. (2016); Shi et al. (2018)
*Coptis chinensis* Franch.	Berberine	*Candida albicans, Candida tropicalis*	Fluconazole	0.03-0.27、0.13-1.0	Shi et al. (2017); Xu et al. (2017)
*Coptis chinensis* Franch., *Hydrastis canadensis* L., *Berberis vulgaris* L.	Berberine chloride	MRSA	Fusidic acid	0.19–0.5	Liang et al. (2014)
*Coptis chinensis* Franch., *Phellodendron amurense* Rupr., *Berberis aristate* DC.	Berberine hydrochloride	*Acinetobacter baumannii*	Tigecycline, Sulbactam, Meropenem and ciprofloxacin	<0.5	Li et al. (2021)
*Coptis chinensis* Franch., *Hydrastis canadensis* L., *Berberis vulgaris* L.	Berberine chloride	*Streptococcus orals*	Penicillin, Clindamycin and Erythromycin	—	Dziedzic et al. (2015); Wultanska et al. (2020); Yong et al. (2020)
*Coptis chinensis* Franch., *Hydrastis canadensis* L.	Berberine chloride	*Clostridium difficile*	Vancomycin	—	-
*Coptis chinensis* Franch.	Berberine hydrochloride	*Candida albicans*	Fluconazole	0.03–0.06	-
*Piper nigrum* L.	Piperine	MRSA	Gentamicin	0.5	Khameneh et al. (2015)
*Sophora alopecuroides* L.	Total alkaloid	*Escherichia coli*	Ciprofloxacin	0.131	Zhou et al. (2013); Pourahmad Jaktaji and Mohammadi, (2018)
*Sophora alopecuroides* L.	Total alkaloid	*Escherichia coli*	Cefotaxime, Ceftazidime	≤0.5	-
*Stephania tetrandra* S. Moore	Tetrandrine	*Candida albicans*	Ketoconazole	—	Zhang et al. (2010)
*Stephania tetrandra* S. Moore	Tetrandrine	MRSA	Cefazolin	0.188–0.625	Zuo et al. (2011)
*Sanguinaria canadensis* L.	Sanguinarine	MRSA	Ampicillin, Oxacillin, Norfloxacin, Ciprofloxacin	0.06–0.75	Obiang-Obounou et al. (2011)

### Phenolics Combined With Antibiotics for Antibacterial Effects

Phenolic compounds are some of the most diverse bioactive secondary metabolites in medicinal plants. They may also be a part of or the main component that contributes to a plants’ bioactivity, with high antibacterial potential (Pinheiro et al., 2018). Phenolic compounds include: epigallocatechin gallate (EGCg), magnolol and honokiol, and eugenol, extracted from *Camellia sinensis* (L.) Kuntze, *Magnolia officinalis* Rehder & E.H.Wilson*,* and *Syzygium aromaticum* (L.) Merr. & L.M.Perry, respectively. Studies have found that they have anti-inflammatory, antibacteria and antioxidant effects (Daglia, 2012). These compounds may also be used to inhibit or kill pathogenic microorganisms (Marino et al., 2001). Researchers have also investigated the application of phenolic compounds with antibacterial drugs in the treatment of bacterial infections.

Hu et al. (Hu et al., 2001; 2002) demonstrated in 2001 that epigallocatechin gallate (EGCg) could be used together with β-lactam antibiotics, such as ampicillin or sulbactam for the treatment of MRSA infection. EGCg can also be combined with carbapenem antibiotics such as imipenem or panipenem in the treatment of MRSA infection, and reverse MRSA resistance. When EGCg is paired with oxytetracycline it has antibacterial effects on MRSA. EGCg at 4 μg/ml showed synergistic and additive effects on six and two clinically tested MRSA strains, respectively, with the FICI from 0.288 to 0.527 (Novy et al., 2013). A study showed that EGCg can further inhibit penicillinase to protect the antibacterial activity of penicillin and ampicillin against penicillinase-producing *S. aureus* (Zhao et al., 2002). It has been reported (Sudano Roccaro et al., 2004) that 50 μg/ml EGCg ( 12 MIC) joined with tetracycline can significantly reduce the MIC of tetracycline against *S. aureus* and exert an obvious antibacterial effect.

Kim et al. (2015) demonstrated that 10 μg/ml magnolol and 25 μg/ml honokiol combined with oxacillin has synergistic effects on MRSA. This application can increase the susceptibility of β-lactam antibiotics to MRSA. *In vivo* and *in vitro* experiments have demonstrated that the survival rate for honokiol combined with fluconazole in the treatment of fluconazole-resistant *C. albicans* infection reached 100%, compared with 20% for honokiol-treated or control group of mice over a period of 5 days (Jin et al., 2010). Sousa Silveira et al. (2020) found that thymol and tetracycline had an anti-*S. aureus* effect. In this study, the results of a fumigation bioassay showed that thymol had an obvious toxic effect on *Drosophila melanogaster* within 48 h of exposure with an EC_50_ (concentration for 50% of maximal effect) value of 17.96 μg/ml. Another study, showed the combination of mupirocin and thymol can enhance the antibacterial activity of mupirocin against MRSA (Kifer et al., 2016). Hemaiswarya and Doble (2009) found that eugenol combined with β-lactam antibiotics such as vancomycin, ampicillin, or oxacillin, had a synergistic antibacterial effect on Gram-negative bacilli. Some scholars (Wang et al., 2018; Dhara and Tripathi, 2020) showed that eugenol combined with colistin enhanced the antibacterial activity of the antibiotics against colistin-resistant *E. coli*, while the combination of eugenol with cefotaxime and ciprofloxacin could resist ESBL-producing quinolone-resistant pathogenic *Enterobacteria*, with FICI ≤0.5. Khan et al. (2019) demonstrated a synergistic effect of low doses (100 μg/ml) of eugenol together with amphotericin B (0.05 μg/ml) against *C. albicans*, with a FICI of 0.27. However, methyl gallate of *Galla Rhois* (*Rhus chinensis* Mill.), or carvacrol and nalidixic acid combination had a synergistic or partial synergistic effect (FICI = 0.31–0.75) on pathogens resistant to nalidixic acid, whereas methyl gallate or carvacrol restored the antibacterial activity of nalidixic acid (Choi et al., 2009).

Bahari et al. (2017) showed that sub-MIC of curcumin combined with azithromycin and gentamicin had a synergistic effect on *P. aeruginosa* PAO1. Moreover, the combination of sub-MIC curcumin and ceftazidime had a synergistic effect on *P. aeruginosa* PAO1 with a FICI of 0.26, and its combination with ciprofloxacin had a FICI of an additive effect (Roudashti et al., 2017). Several studies (Kaur et al., 2018; Itzia Azucena et al., 2019; Sundaramoorthy et al., 2020) showed that curcumin itself did not affect bacterial growth, but when combined with ceftazidime could resist enterotoxin *E. coli* infection. When combined with salicylate and colistin, curcumin could reduce the biological load of colisin-resistant *E. coli* U3790 and *K. pneumoniae* BC936. In addition, curcumin has a synergistic antibacterial effect on *A. baumannii* when paired with colistin. In another study, Wang et al. (Wang et al., 2020) demonstrated that the combination of 12 MIC bisdemethoxycurcumin and 12 MIC gentamicin had a significant synergistic effect on MRSA and a partial synergistic effect with oxacillin or a β-lactam antibiotic.

Abu El-Wafa et al. (2020) showed that the combination of phenolic extracts of pomegranate (*Punica granatum* L.) and rosemary (*Rosmarinus officinalis* L.) with piperacillin, ceftazidime, imipenem, gentamicin, and levofloxacin was effective in treating against *P. aeruginosa* PS-1 and exhibited a synergistic effect (FICI ≤0.5), which radically reduced the MIC of *P. aeruginosa*. Liu et al. (2016) found that the combination of salvianolic acid salt in *Salvia miltiorrhiza* (*Salvia miltiorrhiza* Bge.) and ampicillin applied to MRSA had the best antibacterial effects, which could also reverse MRSA resistance. Table 3 lists the antibacterial effects of the above phenolic compounds combined with antibiotics.

**TABLE 3:** Summary of phenolic compounds in combination with antibiotics.

Species Name	Active ingredients	Drug resistant strains	Combination antibiotics	FICI	References
*Camellia sinensis* (L.) Kuntze	Epigallocatechin gallate	MRSA	Ampicillin, Sulbactam	0.19–0.56	(Hu et al., 2001; 2002)
*Camellia sinensis* (L.) Kuntze	Epigallocatechin gallate	MRSA	Imipenem, Panipenem	≤0.5	-
*Camellia sinensis* (L.) Kuntze	Epigallocatechin gallate	MRSA	Oxytetracycline	0.288–0.527	Novy et al. (2013)
*Camellia sinensis* (L.) Kuntze	Epigallocatechin gallate	*Staphylococcus aureus*	Penicillin, Ampicillin	≤0.5	Zhao et al. (2002)
*Camellia sinensis* (L.) Kuntze	Epigallocatechin gallate	*Staphylococcus aureus*	Tetracycline	—	Sudano Roccaro et al. (2004)
*Magnolia officinalis* Rehder & E.H.Wilson	Magnolol and Honokiol	MRSA	Oxacillin	≤0.5	Kim et al. (2015)
*Magnolia officinalis* Rehder & E.H.Wilson	Honokiol	*Candida albicans*	Fluconazole	0.125–0.5	Jin et al. (2010)
*Thymus vulgaris* L., *Origanum vulgare* L.	Thymol	*Staphylococcus aureus*	Tetracycline	—	Sousa Silveira et al. (2020)
*Thymus vulgaris* L., *Origanum vulgare* L.	Thymol	MRSA	Mupirocin	0.36–0.51	Kifer et al. (2016)
*Eugenia cayophyllata* Thunb., *Syzygium aromaticum* (L.) Merr. & L.M.Perry	Eugenol	Gram-negative bacilli	Vancomycin, Ampicillin, Oxacillin	—	Hemaiswarya and Doble, (2009)
*Eugenia cayophyllata* Thunb., *Syzygium aromaticum* (L.) Merr. & L.M.Perry	Eugenol	*Escherichia coli*	Colistin	0.375–0.5	Wang et al. (2018); Dhara and Tripathi, (2020)
*Eugenia cayophyllata* Thunb., *Syzygium aromaticum* (L.) Merr. & L.M.Perry, *Ocimum gratissimum* L.	Eugenol	*Enterobacter*	Cefotaxime, ciprofloxacin	0.08–0.5	-
*Eugenia cayophyllata* Thunb., *Syzygium aromaticum* (L.) Merr. & L.M.Perry	Eugenol	*Candida albicans*	Amphotericin B	0.27	Khan et al. (2019)
*Rhus chinensis* Mill.	Methyl gallate	Nalidixic acid resistant pathogens	Nalidixic acid	0.12–0.31	Choi et al. (2009)
*Curcuma longa* L.	Curcumin	*Pseudomonas aeruginosa*	Azithromycin, Gentamicin	0.25、0.37	Bahari et al. (2017)
*Curcuma longa* L.	Curcumin	*Pseudomonas aeruginosa*	Ceftazidime	0.26	Roudashti et al. (2017)
*Curcuma longa* L.	Curcumin	*Escherichia coli*	Ceftazidime	—	Kaur et al. (2018); Itzia Azucena et al. (2019); Sundaramoorthy et al. (2020)
*Curcuma longa* L.	Curcumin	*Escherichia coli, Klebsiella pneumoniae*	Colistin	0.03–0.5	-
*Curcuma longa* L.	Curcumin	*Acinetobacter baumannii*	Colistin	0.29	-
*Curcuma longa* L.	Bisdemethoxycurcumin	MRSA	Gentamicin, oxacillin	<0.1	Wang et al. (2020)
*Rosmarinus officinalis* L., *Salvia Rosmarinus* Spenn., *Punica granatum* L.	Phenols	*Pseudomonas aeruginosa*	Piperacillin, Ceftazidime, Imipenem, Gentamicin, Levofloxacin	≤0.5	Abu El-Wafa et al. (2020)
*Salvia miltiorrhiza* Bge.	Salvianolate	MRSA	Ampicillin	0.375	Liu et al. (2016)

### Quinones Combined With Antibiotics for Antibacterial Effects

Quinone compounds in TCM can be divided into four types: benzoquinone, naphthoquinone, phenanthrene quinone, and anthraquinone. Anthraquinone and naphthoquinone are widely used in antibacterial treatment. Anthraquinone compounds from various plants were reported to have antibacterial activity (Novais et al., 2018) and anti-inflammatory, antifungal and antiviral effects (Li and Jiang, 2018). Naphthoquinone and naphthoquinone derivatives (Janeczko et al., 2016) were also reported to have antibacterial activity. Rhein extracted from *Rheum palmatum* L., resveratrol from the rhizome of *Polygonum cuspidatum* Sieb. et Zucc.*,* and cryptotanshinone from *Salvia miltiorrhiza* Bge. are quinones. Quinone compounds in combination with antibiotics have been developed as a new measure for treating antibiotic resistance.

Joung et al. (2012) demonstrated that the FICI of rhein combined with ampicillin or oxacillin for all MRSA strains was 0.28–1 and 0.18–1, respectively and showed a synergistic or partial synergistic effect. Cannatelli et al. (2018) reported that resveratrol had no obvious intrinsic antibacterial activity but displayed synergistic effects with colistin on colistin-resistant Gram-negative bacilli of different species. Resveratrol oxide combined with vancomycin and ciprofloxacin had a synergistic effect on MRSA. It was partially additive or synergistic for the combination of resveratrol oxide with ampicillin, oxacillin, and norfloxacin. These combinations completely inhibited the growth of bacteria after 24 h (Joung et al., 2015). Studies have found that hypericin and β-lactam antibiotics such as oxacillin have anti-MRSA ability (Wang et al., 2019). Cha et al. (2014) demonstrated that cryptotanshinone combined with ampicillin, oxacillin, or vancomycin had synergistic effects on methicillin-resistant and vancomycin-resistant *S. aureus* and greatly inhibited the growth of bacteria. In addition, cryptotanshinone, together with gentamicin and streptomycin at safe doses (gentamicin ≤12 μg/ml and streptomycin ≤20 μg/ml) had a synergistic antibacterial effect on *S. aureus*. It reduced the resistance of aminoglycoside antibiotics to drug-resistant *S. aureus*, while the combination of cryptotanshinone with fosfomycin showed synergistic effect on fosfomycin-sensitive and fosfomycin-resistant *S. aureus* (FICI, 0.3125–0.375) (Teng et al., 2018; Ruan et al., 2020). Table 4 lists the antibacterial effects of the above quinones in combination with antibiotics.

**TABLE 4:** Summary of quinone compounds in combination with antibiotics.

Species Name	Active ingredients	Drug resistant strains	Combination antibiotics	FICI	References
*Rheum palmatum* L.	Rhein	MRSA	Ampicillin, Oxacillin	0.28-1、0.18-1.0	Joung et al. (2012)
*Vitis vinifera* L., *Morus alba* L.	Resveratrol	Gram-negative bacteria	Colistin	≤0.5	Cannatelli et al. (2018)
*Morus alba* L.	Oxyresveratrol	MRSA	Vancomycin, Ciprofloxacin	0.375	Joung et al. (2015)
*Hypericum perforatum* L.	Hypericin	MRSA	Oxacillin	0.1–0.16	Wang et al. (2019)
*Salvia miltiorrhiza* Bge.	Cryptotanshinone	*Staphylococcus aureus*	Ampicillin, Oxacillin, vancomycin	≤0.5	Cha et al. (2014)
*Salvia miltiorrhiza* Bge.	Cryptotanshinone	*Staphylococcus aureus*	Gentamicin, Streptomycin	0.25-0.5, 0.375-0.5	Teng et al. (2018); Ruan et al. (2020)
*Salvia miltiorrhiza* Bge.	Cryptotanshinone	*Staphylococcus aureus*	Fosfomycin	0.3125–0.375	-

### Other Compounds Combined With Antibiotics for Antibacterial Effects

Lu et al. (2013) demonstrated that sodium new houttuyfonate could be synergistic with cephalosporin, meropenem, oxacillin, and netilmicin against MRSA infection. The median FIC of the checkerboard method was 0.38, 0.38, 0.25, and 0.38, respectively. Several studies (Jiang et al., 2011; Li et al., 2011; Wei et al., 2020) reported that artesunate combined with oxacillin and ampicillin had a synergistic antibacterial effect on MRSA. Combined with β-lactam antibiotics such as ampicillin, artesunate could also inhibit *E. coli* infection and enhance the antibacterial activity of fluoroquinolones against multidrug-resistant *E. coli*. The combination of 3-benzylchroman derivatives from the Chinese drug, *Caesalpinia sappan* L., with the aminoglycoside antibiotic can also be effective against MRSA. Morin, and trans-cinnamaldehyde combined with oxacillin has shown a synergistic effect against MRSA and potential for reversing the drug resistance of MRSA. M*agnolia officinalis* (*Magnolia officinalis* Rehder & E.H.Wilson) and *Verbena* (*Verbena officinalis* L.) extracts combined with oxacillin have otherwise showed a synergistic effect with partial efficacy against MRSA infection, where the colony number decreased by 3log10 cfu/mL (DPS-1 and DPS-3) after a treatment with a combination of 12 MIC morin and 12 MIC oxacillin for 24 h (Zuo et al., 2014; Mun et al., 2015; Kuok et al., 2017; Wang et al., 2021). Some scholars (Vazquez et al., 2016; Buommino et al., 2021) demonstrated that the pairing of rosin acid and oxacillin increased the susceptibility of methicillin-resistant *Staphylococcus pseudo intermediate* to oxacillin. Conversely, carnosic acid and gentamicin had obvious synergistic effects of bactericidal and bacteriostasis on clinical isolates of multidrug-resistant MRSA, while 4 μg/ml gentamicin combined with 4 μg/ml carnosic acid showed a 100% inhibition on bacterial growth. Fatemi et al. (2020) found that methanol extract of *Salvia chorassanica* (*Salvia chorassanica* Bunge) and *Artemisia khorassanica* (*Artemisia oliveriana* J. Gay ex Besser) synergically enhanced the susceptibility of multidrug-resistant *A. baumannii* with amikacin and imipenem. In addition, the combination of zingerone and ciprofloxacin significantly inhibited the formation of *P. aeruginosa* PAO1 biofilm and played an antibacterial role. *Stephania suberosa* Forman extract (2 mg/ml) in combination with ampicillin (0.15 μg/ml) had a significant effect on the treatment of MRSA infection and significantly reduced the dosage of ampicillin from >512 μg/ml (used alone) to 0.15 μg/ml (combined with the extract) (Kumar et al., 2013; Yothin Teethaisong 2014). Table 5 lists the antibacterial effects of other active ingredients mentioned above in combination with antibiotics.

**TABLE 5:** Summary of other compounds in combination with antibiotics.

Species Name	Active ingredients	Drug resistant strains	Combination antibiotics	FICI	References
*Houttuynia cordata* Thumb.	Sodium new houttuyfonate.	MRSA	Cephalosporin, Meropenem, Oxacillin, Netilmicin	0.25–0.38	Lu et al. (2013)
*Artemisia annua* L.	Artesunate	MRSA	Oxacillin, Ampicillin	<0.37	Jiang et al. (2011); Li et al. (2011); Wei et al. (2020)
*Artemisia annua* L.	Artesunate	*Escherichia coli*	Ampicillin	≤0.5	-
*Artemisia annua* L.	Artesunate	*Escherichia coli*	Fluoroquinolone antibiotics	0.12–0.33	-
*Caesalpinia sappan* L.	3-Benzylchroman derivatives	MRSA	Aminoglycoside antibiotics	0.375–0.5	Zuo et al. (2014); Mun et al. (2015); Kuok et al. (2017); Wang et al. (2021)
*Magnolia officinalis* Rehder & E.H.Wilson, *Verbena officinalis* L., *Cinnamomum cassia* Presl	Morin, Tiliroside, Pinoresinol, Trans-Cinnamaldehyde	MRSA	Oxacillin	0.28–0.75	-
*Pinus caribaea* Morelet	Abietic acid	*Pseudo intermediate staphylococcus*	Oxacillin	0.375	-
*Rosmarinus officinalis* L.	Carnosic acid	MRSA	Gentamicin	0.5	Vazquez et al. (2016); Buommino et al. (2021)
*Salvia chorassanica* Bunge, *Artemisia khorassanica* Podlech, *Artemisia oliveriana* J.Gay ex Besser	Methanol extracts	*Acinetobacter baumannii*	Amikacin, Imipenem	0.185-0.625、0.18-0.37	-
*Zingiber officinale* Rosc.	Zingerone	*Pseudomonas aeruginosa*	Ciprofloxacin	—	Kumar et al. (2013); Yothin Teethaisong (2014)
*Stephania suberosa* Forman	Cepharanthine	MRSA	Ampicillin	<0.5	-

## Conclusion

TCM has great antibacterial potential, with low toxicity, low drug resistance, and abundant resources. With further research on the mechanism of bacterial drug resistance and the continuous progress in the extraction technology of effective ingredients of TCM, the combined application of various active ingredients or compounds of TCM and antibiotics in the control of bacterial or drug-resistant bacteria infection has been widely studied. The active ingredients of TCM act as synergists by enhancing the antibacterial activity, improve the therapeutic effect and reduce the dosage of antibiotics and adverse reactions. At present, all studies on antibacterial or bacteriostatic effects from the combination of active ingredients of TCM and antibiotics have been conducted *in vitro*. There is insufficient evidence to prove the effectiveness, stability, selective toxicity, and targeted availability of these combinations in the human body. Therefore, further *in vivo* studies and animal models are needed. This paper summarises the interaction between different compounds of TCM, such as flavonoids, alkaloids, phenols and quinones, with antibiotics in the fight against drug-resistant bacteria. Using different active TCM ingredients with the same antibiotic, has a synergistic effect on drug-resistant bacteria. The same TCM ingredient can also have a synergistic antibacterial effect with different antibiotics. The above studies found that the combination of quercetin and berberine with antibiotics yielded good synergistic antibacterial effects and a broad antibacterial spectrum. Therefore, as the most researched active ingredients of TCM with strong antibacterial effects, flavonoids and alkaloids will be promising antibacterial choices when used in combination with antibiotics. This provides a new avenue to solve the problem of bacterial resistance through TCM and an important theoretical basis for finding alternative methods to counteract resistant bacteria. The combined use of TCM and antibiotics has become a new and alternative trend for antibacterial treatment. In the face of the current drug resistance crisis and the dilemma of new drug research and development, finding a more effective and safer alternative for the treatment of drug-resistant bacterial infection is crucial. The in-depth study of the synergistic antibacterial effect and synergistic mechanism of the combination of active components of TCM and antibiotics *in vivo,* may become an important research direction in the future.
